# Effect of Non-Lethal Selection on Spontaneous Revertants of Frameshift Mutations: The *Escherichia coli* *hisF* Case

**DOI:** 10.3390/microorganisms10040692

**Published:** 2022-03-23

**Authors:** Sara Del Duca, Anna Maria Puglia, Vito Calderone, Marco Bazzicalupo, Renato Fani

**Affiliations:** 1 Laboratory of Microbial and Molecular Evolution, Department of Biology, University of Florence, Via Madonna del Piano 6, 50019 Sesto Fiorentino, Italy; sara.delduca@unifi.it (S.D.D.); marco.bazzicalupo@unifi.it (M.B.); 2 Laboratory of Molecular Microbiology and Biotechnology, STEBICEF Department, University of Palermo, Viale delle Scienze Ed. 16, 90128 Palermo, Italy; a.maria.puglia@unipa.it; 3 Magnetic Resonance Center (CERM)/Interuniversity Consortium for Magnetic Resonance of Metallo Proteins (CIRMMP), Via L. Sacconi 6, 50019 Sesto Fiorentino, Italy; vito.calderone@unifi.it; 4 Department of Chemistry “Ugo Schiff”, University of Florence, Via della Lastruccia 3, 50019 Sesto Fiorentino, Italy

**Keywords:** directed-evolution experiment, reverse mutation, protein structure

## Abstract

Microorganisms possess the potential to adapt to fluctuations in environmental parameters, and their evolution is driven by the continuous generation of mutations. The reversion of auxotrophic mutations has been widely studied; however, little is known about the reversion of frameshift mutations resulting in amino acid auxotrophy and on the structure and functioning of the protein encoded by the revertant mutated gene. The aims of this work were to analyze the appearance of reverse mutations over time and under different selective pressures and to investigate revertant enzymes’ three-dimensional structures and their correlation with a different growth ability. *Escherichia coli* FB182 strain, carrying the *hisF892* single nucleotide deletion resulting in histidine auxotrophy, was subjected to different selective pressures, and revertant mutants were isolated and characterized. The obtained results allowed us to identify different indels of different lengths located in different positions in the *hisF* gene, and relations with the incubation time and the selective pressure applied were observed. Moreover, the structure of the different mutant proteins was consistent with the respective revertant ability to grow in absence of histidine, highlighting a correlation between the mutations and the catalytic activity of the mutated HisF enzyme.

## 1. Introduction

Microorganisms possess the potential to adapt to fluctuations in the environmental parameters of the niche they live in. They can develop novel metabolic functions through the activation of cryptic and silent genes, the selection of mutations in regulatory and/or structural genes, and also by the acquisition of new genes by horizontal transfer mechanisms [1,2]. In addition, gaining a lost metabolic function can also be achieved through the restoration of prototrophy in microorganisms harboring mutation(s) in one (or more) gene(s) involved in a given metabolic pathway, such as amino acid biosynthesis.

Most of the studies concerning the origin of mutations have been largely debated since the last decades of the last century. Cairns and his collaborators [3] showed that mutations can arise in apparently static bacterial populations when subjected to nonlethal selective pressure, and evidenced that only selected for mutations appeared in a population during selection [4,5]. Indeed, selection allows beneficial mutations to increase in frequency, while neutral or deleterious types are easily missed [6]. This phenomenon was called “adaptive mutation”, and several models for the generation of these induced mutations have been proposed [3,7,8,9]. The most complete body of evidence in support of adaptive mutation has involved *Escherichia coli* mutants defective in lactose utilization (strain FC40) [10]; when subjected to extended incubation on minimal lactose medium, Lac^+^ revertant colonies arose and were isolated and characterized [11]. Today, it is well known that evolution by natural selection is driven by the continuous generation of mutations, and microorganisms’ enormous potential for adaptation may also explain why antibiotic resistance and virulence evolve so quickly in pathogenic strains [12,13]. Moreover, selective pressure on microbial amino acid auxotrophic strains can also be designed to evolve synthetic strains that can live with unnatural amino acids [14,15,16,17].

Concerning the reversion of amino acid auxotrophy, this phenomenon has been especially studied in the enterobacterium *E. coli* K12, harboring missense mutations in either *trpA* (*trpA46*) or *trpB* (*trpB9578*) genes (which encode the α and β subunits of tryptophan synthase) [4], or nonsense (ochre) mutations in *trpE* (*trpE65*) (encoding a subunit of anthranilate synthase), *tyrA* (*tyrA14*) (encoding a T-protein required for tyrosine biosynthesis) [18] or *argE* (*argE3*) (encoding a deacetylase involved in arginine biosynthesis) [19]. Moreover, reversion in mutations in the histidine operon of *Salmonella typhimurium* was also studied, either by the presence of mutagens in the culture medium, such as in the Ames mutagenicity assay used to detect chemical substances that can produce genetic damage [20], or in conditions of histidine starvation. In this case, two mutants altered in the *hisG* gene (*hisG46* and *hisG428*), encoding the first enzyme of the histidine biosynthesis (i.e., the ATP phosphoribosyl transferase) were used, carrying a missense and a nonsense (ochre) mutation, respectively. Both mutants restored the His^+^ phenotype by mutations in either intra- or extragenic regions [21,22].

To the best of our knowledge, very little is known about the reversion of frameshift mutations resulting in amino acid auxotrophy and the correlation with the structure of the protein encoded by the mutated gene, as well as the growth rate in the absence of the amino acid whose biosynthetic pathway has been altered. For this reason, we investigated the different mutations restoring the prototrophy in an auxotrophic *E. coli* mutant (strain FB182 [23]) harboring the *hisF892* single nucleotide deletion [24], causing a frame shift and resulting in the inability of *E. coli* cells to grow in the absence of histidine. The *hisF* gene, involved in the histidine biosynthetic pathway, was chosen as the subject of this study since it encodes a protein whose structure has been clearly elucidated in recent years, especially in the thermophilic bacterium *Thermotoga maritima*. This enzyme belongs to the TIM barrel family, and it is constituted by eight β-strands, each of which is separated by an α-helix. Since this (β/α)_8_ barrel structure is probably the most common enzyme fold and can be obtained by different amino acid sequences [25,26], the aims of this work were to test (i) whether the alteration of this three-dimensional structure might be compensated by different reverse mutations, which might restore—totally or partially—the correct three-dimensional structure with a different ability to allow growth in the absence of histidine, and (ii) whether different reverse mutations might appear over time and/or depending on the selective pressure applied.

## 2. Materials and Methods

### 2.1. Bacterial Strains 

The *E. coli* FB8 strain (wild-type *E. coli* K12 UTH1038 [27]) and *E. coli* FB182 (*hisF892*) strain [23] were used in the present work. *E. coli* FB182 carries a single nucleotide deletion in position 718 or 719 of the *hisF* gene, causing a frame shift and the formation of a premature stop codon, resulting in a shorter (243 aa vs. 258 aa of the wild-type *E. coli* HisF protein) and non-functioning enzyme [24].

The list of the 67 *E. coli* FB182 HisF^+^ revertant strains obtained in this work is reported in Table 1.

### 2.2. Directed-Evolution Experiment and Isolation of HisF^+^ Revertants

*E. coli* FB182 HisF^+^ revertants were obtained as follows:

(i) *E. coli* FB182 cells were grown overnight at 37 °C with shaking (150 rpm) in minimal medium Davis (MMD) [28] ((NH_4_)_2_SO_4_ 1 g/L; K_2_HPO_4_ 7 g/L; KH_2_PO_4_ 2 g/L; Na_3_-citrate·2H_2_O 0.5 g/L; MgSO_4_·7H_2_O 0.1 g/L; pH 7.2) with glucose 1% and histidine 25 µg/mL.
(ii) The optical density (O.D._600_) of the culture was measured and the culture was diluted to O.D._600_ 0.1 in a final volume of 50 mL of MMD containing glucose 1% and histidine 25 µg/mL.
(iii) The culture was then incubated at 37 °C with shaking (150 rpm); at the end of the log phase, cells were centrifuged, washed twice in saline solution (NaCl 0.9% *w*/*v*) and then spread on 100 mL MMD plates containing agar 1.6% and glucose 1% in the absence of histidine (three plates), or in the presence of histidine 0.3 µg/mL (three plates) or 1 µg/mL (three plates). 100 µL of 10^−5^ and 10^−6^ dilutions were plated on LB agar (Lysogeny Broth) [29] (NaCl 10 g/L, yeast extract 5 g/L, tryptone 10 g/L, agar 16 g/L), to evaluate the cells’ vital titer.
(iv) Vital titer plates were incubated at 37 °C overnight. Selective pressure plates were incubated at 37 °C for 30 days, and the appearance of HisF^+^ revertants was checked daily.
(v) Three different experiments were carried out in triplicate.
(vi) HisF^+^ revertants were tested for their ability to grow in the total absence or in low concentrations of histidine through streaking on MMD plates containing glucose 1% and histidine 0, 0.3, 1 µg/mL.

Moreover, the number of viable cells plated on MMD in the absence or in the presence of 0.3 and 1 µg/mL of histidine was determined during the entire experiments as follows: every day, a portion of 1 cm diameter was picked up from each of the selective plates. The agar was then washed three times with saline solution (NaCl 0.9% *w*/*v*) for 30 s in order to recover the *E. coli* FB182 cells from the surface. Cells were then spread on MMD plates containing histidine (25 µg/mL) and incubated at 37 °C overnight, and the vital titer was calculated. 

### 2.3. PCR Amplification and Sequencing

The nucleotide sequence of the *E. coli* K12 MG1655 (accession NC_000913) histidine operon was obtained from the NCBI database. Based on this sequence, primers were designed to anneal externally to *hisF* gene; the primers used in this study are coli_hisF_ext FW (5′-GCGGCGTAATAGTTGGTCG-3′) and coli_hisF_ext REV (5′-TCTAAGGCTTCCGGGTTCAT-3′), which anneal 61 bp upstream and 121 bp downstream of the *hisF* gene (777 bp), respectively, generating an amplicon of 959 bp. For the amplification of the HisF^+^ revertant *hisF* gene, thermal lysates were used as template. The PCR reaction was carried out in a total volume of 20 µL containing: DreamTaq buffer 1× (Thermo Fisher Scientific, Waltham, MA, USA), 0.25 mM of dNTPs, 0.2 µM of each primer, 1 U of DreamTaq polymerase (Thermo Fisher Scientific) and 1 µL of lysate. The amplification reaction was set as follows: 5 min at 95 °C for the initial denaturation, 35 cycles of 95 °C for 30 s, 59 °C for 45 s and 72 °C for 1 min, and the final extension at 72 °C for 10 min. PCR products were analyzed through 0.8% *w*/*v* agarose gel electrophoresis with 1X ethidium bromide solution in 1× TAE buffer.

PCR products were diluted 1:5 in a final volume of 5 µL and purified with ExoSAP-IT™ Express PCR Product Cleanup kit (Applied Biosystems, Waltham, MA, USA) following manufacturer instructions. A sequencing reaction was performed using the BigDye™ Terminator v3.1 Cycle Sequencing kit (Applied Biosystems) in a total volume of 10 µL, containing: 1 µL of BigDye™ Terminator v3.1 Ready Reaction Mix, Sequencing Buffer 1× and 0.32 µM of primer forward or primer reverse. The sequencing reaction was set as follows: 3 min at 96 °C for the initial denaturation, 25 cycles of 96 °C for 10 s, 50 °C for 5 s and 60 °C for 4 min. Sequencing reactions were purified with BigDye XTerminator™ (Applied Biosystems) following manufacturer instructions. Capillary electrophoresis was performed in a SeqStudio Genetic Analyzer (Applied Biosystems). Obtained sequences were analyzed using BioEdit [30].

### 2.4. Prediction of Protein Three-Dimensional Structure

The three-dimensional structure of the HisF proteins encoded by the *E. coli* FB182 HisF^+^ revertants was predicted with RoseTTAFold software [31] through a comparative modeling approach. The template used was *E. coli* K12 HisF protein, predicted through AlphaFold2 [32] and available on the AlphaFold2 Protein Structure Database (accession number B1X6W2). Three-dimensional structures were superposed and the relevant root-mean-square deviations (RMSD) on all backbone atoms were calculated using UCSF Chimera v. 1.16 [33]. 

### 2.5. His^+^ Revertant Growth Curves and Statistical Analyses

*E. coli* FB182 HisF^+^ revertant growth curves were performed in microplates. Cells were grown overnight at 37 °C with shaking (150 rpm) in LB. The next morning, cell cultures were washed twice in saline solution (NaCl 0.9% *w*/*v*) and diluted to a starting O.D._600_ of 0.01 in MMD supplemented with glucose 1% and histidine 0.3, 1, 25 μg/mL when required. Cells were then incubated at 37 °C and the O.D._600_ measures were taken every hour for 48 h using the Infinite M Nano (Tecan, Männedorf, Switzerland) microplate reader. Each curve was performed in triplicate. 

The instantaneous growth rate of bacterial cultures was calculated according to Widdel [34]. Maximum growth rate (µ_MAX_) was chosen to compare a specific mutant growth in the absence and presence of histidine in the culture medium. The average µ_MAX_ and the relative standard error were calculated since every growth curve was performed in triplicate; then, the ratio between the µ_MAX_ in the absence and presence of histidine was obtained. The same procedure was applied for the calculation of the ratio between the areas under the growth curve (AUC), obtained according to the chained trapezoidal rule [35].

An analysis of variance (ANOVA) using Tukey’s pairwise test was performed on the two ratios (between µ_MAX_ (r-µ_MAX_) in the absence and presence of histidine, and AUC (r-AUC) in the absence and presence of histidine) among the different cell populations.

## 3. Results and Discussion

### 3.1. Isolation of E. coli FB182 HisF^+^ Revertants

HisF^+^ revertants from the *E. coli* strain FB182 were isolated as described in Materials and Methods on MMD plates in the absence of histidine or in the presence of two different histidine concentrations (i.e., 0.3 and 1 µg/mL, respectively). At the end of the experiments a total of 67 His^+^ colonies were found on all the selective plates and recovered. In particular, as shown in Table 1:

(i) 11 HisF^+^ colonies were isolated on MMD in the absence of histidine out of 3 × 10^9^ viable cells plated;
(ii) 20 HisF^+^ colonies were isolated on MMD in the presence of 0.3 µg/mL histidine out of 3 × 10^9^ viable cells plated;
(iii) 36 HisF^+^ colonies were isolated on MMD in the presence of 1 µg/mL histidine out of 3 × 10^9^ viable cells plated.

### 3.2. Genetic Characterization of His^+^ Revertants

In order to identify the type and the localization, within the *E. coli* FB182 *hisF* gene, of the mutation(s) responsible for the HisF^+^ phenotype, a DNA fragment containing the entire *hisF* gene and its surrounding regions was amplified from each of the 67 HisF^+^ revertants via PCR, as described in Materials and Methods. An amplicon of the expected size (i.e., 959 bp) was obtained from each of them (not shown), suggesting that no large rearrangement occurred in the *hisF* gene of any of the 67 revertants.

**Figure 1:**
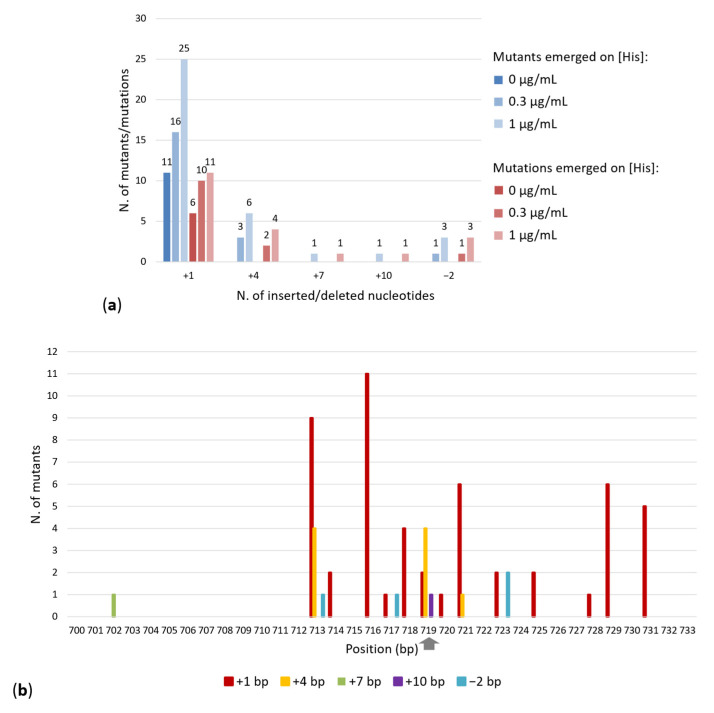
(**a**) Distribution of *E. coli* FB182 HisF^+^ revertants (in different shades of blue) and of the characterized mutations (in different shades of red). (**b**) Spatial distribution of mutations causing reversion in *hisF* gene. When a mutation could have occurred in multiple positions, the position of the last nucleotide(s) of the series is reported. The grey arrow points the position of *E. coli* FB182 mutation.

The nucleotide sequence of each amplicon was then determined and compared with that of both the *E. coli* wild-type strain and mutant *E. coli* FB182. The data obtained are shown in Table 2 and in Figure 1A, whose analysis revealed that—as it might be expected since the point mutation affecting the function of *hisF* gene in the mutant strain *E. coli* FB182 is a 1 bp deletion—two main types of genetic rearrangements occurred, i.e., either insertions or deletions (indels) of different lengths. In particular:

(i) Insertion of four different length (+1,+4, +7, +10 bp) occurred;
(ii) Just one type of deletion took place (−2 bp); no deletions longer than 2 bp were detected;
(iii) The highest number of mutants (52 out of 67 corresponding to 78%) exhibit a +1 bp insertion; nine mutants exhibit a +4 bp insertion; 1 mutant exhibited a +7 bp and 1 a +10 bp insertion;
(iv) Four mutants showed a −2 bp deletion;
(v) Fifteen different types of +1 insertions occurred (one in an identical position, five upstream and eight downstream of the *E. coli* FB182 deletion). Moreover, a single strain (ID 15) harbors, aside from a +1 insertion located upstream of the *E. coli* FB182 deletion, a single nucleotide substitution. To simplify, this strain was considered to be part of the +1 insertions group.
(vi) Five different types of +4 insertions took place (two upstream and three downstream of the *E. coli* FB182 deletion).

As might be expected, all the insertions and deletions detected are in close proximity to the original mutation. Concerning the +1 insertions, a very small number (2/52, 3.8%) of +1 bp insertions fell in this position. Indeed, the highest number of +1 insertions occurred between positions 710 and 716 (a total of 22), corresponding to 33% of the entire panel of mutants (Figure 1B). 

### 3.3. Spatial Distribution of Mutations Causing HisF^+^ Reversion

The spatial distribution of the indels determining the reversion of the *hisF892* mutation is shown in Figure 1B. As can be observed, the distribution spans from position 702 to 731. No difference between the number of the mutation reversion falling upstream or downstream of the *hisF892* mutation was found. This might be expected; indeed, indels falling in proximity to the *E. coli* FB182 −1 bp deletion might change just a few numbers of residues which, in turn, might not alter the three-dimensional structure of the protein. On the other hand, a reversion occurring distantly from the original mutation would involve a number of amino acids sufficiently high to affect the correct folding of the last (β/α) module, thus interfering with the function of the HisF protein. Indeed, the region spanning from nucleotide 601 to nucleotide 729 (comprising the β-sheets/α-helices β-7, α-7, β-8, α-8) corresponds to the phosphate-binding domain (Table 2) [36]. Indeed, for HisF, it is known that both β/α loops 3 and 4 and β/α loops 7 and 8 contain phosphate-binding motifs, required by the nature of its biphosphate substrate (PRFAR) [37,38,39]. This motif is found in the C-terminal halves of a number of other TIM barrel enzymes (but not in the N-terminal halves) [40] and it is the most conserved region in many (β/α)_8_ barrels, including the *E. coli* HisF and HisA [36]. All the mutations obtained in this work fell in the HisF C-terminal phosphate-binding site. The prediction of the amino acid sequence of the *E. coli* FB182 HisF protein by inserting a single nucleotide (A, G, T, or C) in each position from 697 to 713 deeply altered the amino acid sequence of the phosphate-binding site (not shown), very likely interfering with the HisF catalytic activity.

### 3.4. Effect of the Strength of Selective Pressure

**Figure 2:**
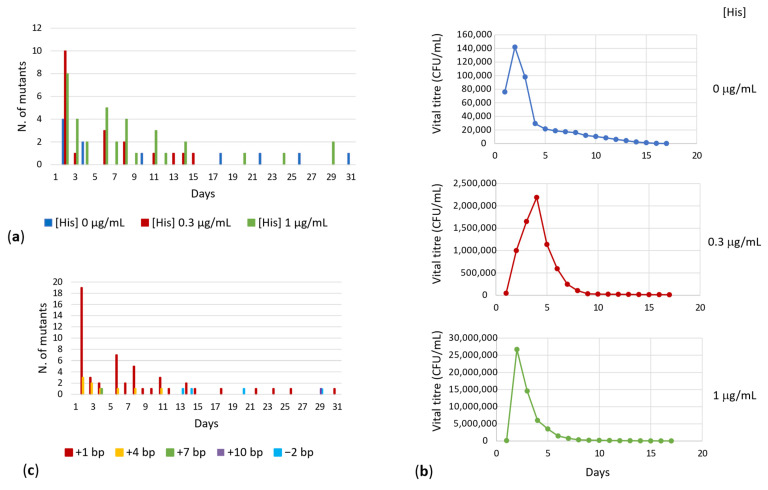
(**a**) Timing of appearance of *E. coli* FB182 HisF^+^ revertants under different selective pressures. (**b**) Survival of *E. coli* FB182 cells under different selective pressure (i.e., different histidine concentrations in the culture medium). (**c**) Timing of appearance of *E. coli* FB182 His^+^ revertants on the basis of the different mutation.

Results concerning the appearance of *E. coli* FB182 HisF^+^ colonies are shown in Figure 2A. As can be observed, the number of revertants increased with the increase in histidine concentration in the growth minimal medium. In other words, the lower the selective pressure (i.e., the higher the histidine concentration in the growth medium), the higher the number of HisF^+^ colonies obtained. This finding suggested that the arise of HisF^+^ colonies may require a residual growth of bacteria enabling DNA replication which, in turn, might allow the accumulation of revertants. On the other hand, plates without histidine still allowed the growth of 11 revertants, indicating that starving cells can induce mutations under stressful conditions [41].

In order to check this idea, the number of viable cells plated under selective pressure was determined throughout the entire experiment, as reported in Materials and Methods. The data obtained are reported in Figure 2B, whose analysis revealed that, after an initial increase in viable titer, the number of viable cells decreased constantly over time. However, a residual number of viable cells was also present after a long period of incubation at 37 °C, even in the absence of histidine.

The largest rearrangements (+4, +7, +10, −2 bp) appeared only on MMD plates containing 0.3 or 1 µg/mL histidine (Figure 1A), suggesting that the mechanisms of mutation under a very strong selective pressure (i.e., the absence of the amino acid) could be different from that under mild selection conditions, not allowing the appearance of large rearrangements. It is not clear, on the basis of the available data, whether these larger rearrangements occurred in a single step or required multiple successive events. 

### 3.5. Effect of Time

The data reported in Figure 2A show the timing of the appearance of *E. coli* FB182 HisF^+^ revertants; the analysis of these data revealed that no revertant appeared in the first 24 h after plating on MMD medium, independently from the applied selective pressure, while the wild-type strain in the same media shows well-grown colonies after 24 h. This result, together with the fact that mutants belonging to the most represented groups of mutations appeared in different days on different plates, suggested that no revertant was present in the bacterial culture before plating on MMD medium. The appearance of His^+^ revertants began after at least 48 h of incubation at 37 °C and the number of HisF^+^ colonies increased over time up to the 31st day. No additional revertant was detected later.

About one-third of the entire panel of HisF^+^ revertants appeared on the second day of incubation at 37 °C. Overall, the longer the rearrangement occurred, the longer the time requested for its appearance. The timing of the appearance of *E. coli* FB182 His^+^ revertants on the basis of the different mutation is reported in Figure 2C. The majority of the most severe reversion mutations appeared after at least 13 days of incubation, suggesting a correlation between the long incubation time and the type of mutation that occurred.

### 3.6. Analysis of the Amino Acid Sequence of the HisF Proteins from HisF^+^ Revertants and Prediction of Tertiary Structure of HisF Proteins from wild-type E. coli and HisF^+^ Revertants

The *hisF* nucleotide sequence from each of the 25 *E. coli* FB182 HisF^+^ revertant groups was translated into the amino acid sequence of the corresponding protein. The data obtained concerning the regions immediately upstream and downstream of the *hisF892* mutation are reported in Table 3, whose analysis revealed that 22 different predicted amino acid sequences were disclosed, since revertants belonging to class 2 and 3 harbor the same amino acid sequence, as well as mutants belonging to classes 4 and 5 and 8 and 11.

**Figure 3:**
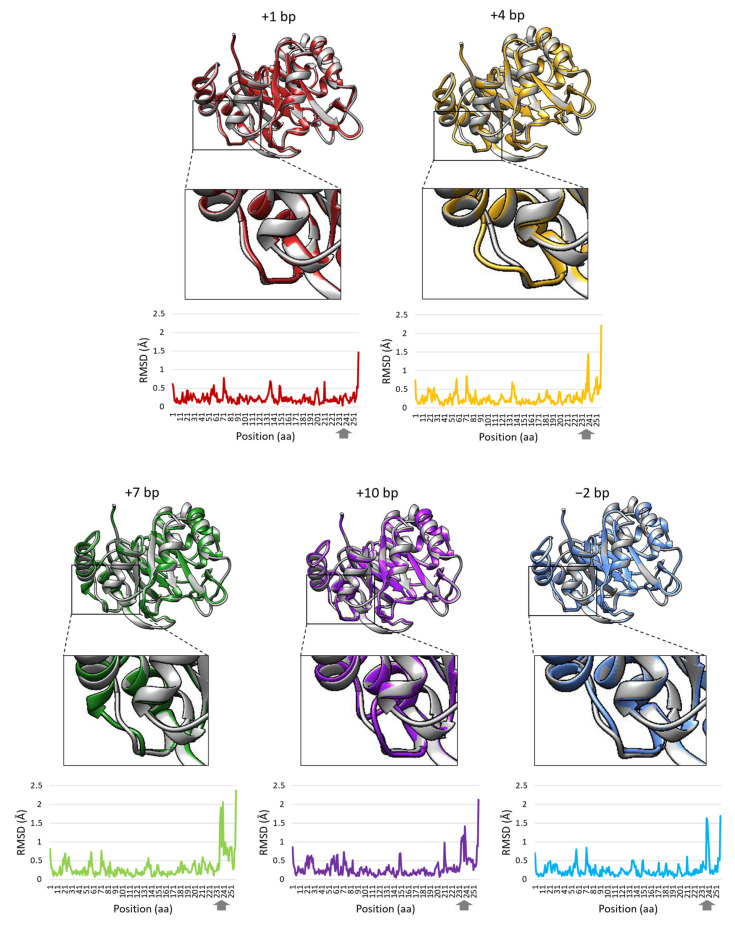
Prediction of the three-dimensional structure of *E. coli* FB182 revertants HisF protein superimposed on the three-dimensional structure of wild-type *E. coli* K12 HisF available on the AlphaFold2 Protein Structure Database (accession number B1X6W2) (in grey). Prediction was performed for one representative of each indels group (+1 bp group: ID 14; +4 bp group: ID 19; +7 bp group: ID 21; +10 bp group: ID 22; −2 bp group: ID 24). Detail of the HisF protein region containing the mutations is reported. RMSD values calculated on all backbone atoms between wild-type *E. coli* K12 HisF and HisF revertants are reported. The grey arrows point the position of mutations.

In order to check whether the mutation(s) that occurred in *E. coli* FB182 HisF^+^ revertants might have either restored the correct or altered the three-dimensional structure of the HisF protein, tertiary structure prediction was carried out as described in Materials and Methods and shown in Figure 3.

The two approaches used for structure prediction were AlphaFold2 and RoseTTAFold; they represent the two most reliable methods available nowadays to accomplish the always-dreamed-of capability of predicting three-dimensional structures, starting from the amino acid sequence [43,44]. In particular, the structure of the wild-type *E. coli* K12 HisF protein was directly downloaded from the AlphaFold2 Database, whereas all the mutant HisF structures were calculated with RoseTTAFold as reported in Materials and Methods. The superposition of the wild-type HisF with each mutant one showed, as expected, a significant structural difference in the region involving the mutations. The calculation of the RMSD between the wild-type and each revertant showed no significant deviation for mutants belonging to the +1 bp insertion group, where the number of residues does not change from the wild-type. Concerning the mutants that imply an increase or a decrease in the number of residues, the RMS deviation in that specific region went beyond 1.4 Å, reaching values beyond 2 Å for mutants belonging to the +7 bp insertion group. Mutants belonging to the +4 bp groups showed an RMSD around 1.5 Å; the mutant with the +10 bp insertion, despite the higher number of nucleotide insertions, showed an RMSD around 1.5 Å, but the perturbation was spread over a larger number of residues (Figure 3). These deviations are clearly visible in Figure 3, where the three-dimensional structure for one representative of each mutation group is reported. The higher the number of residue insertions, the higher the structural perturbation of the backbone in the C-terminal region. Despite the variations being located in a restricted residue range, this region might be involved in the catalytic activity of the protein. As already outlined in Paragraph 3.3, this region is critical to the catalytic activity of HisF because it binds a second phosphate; it is plausible that even modest rearrangements of the structure in this specific region could, for example, cause a decreased binding affinity for this phosphate, thus affecting the catalytic efficiency of the enzyme. 

An important structural parameter that would be useful to characterize in deeper detail the difference between wild-type and mutants is indeed the B-factors distribution. Their value indicates the degree of mobility of each atom and would allow for speculating about the possible implications and effects caused by the mutations. Unfortunately, in this case, no B-factors or no significant B-factors are available since the models used are not experimental.

Moreover, as a control, all the structures predicted using the comparative modeling approach in RoseTTAFold were calculated also using the ab initio approach; the results obtained by performing the calculations with the model obtained in such a way were indeed superposable to those obtained with the comparative modeling approach.

### 3.7. Correlation of Reversion Mutation Occurred with the Ability to Grow in the Absence of Histidine

Since the 67 revertants were isolated on MMD under different selective pressures, there is no telling if the revertants that appeared on MMD plates under low selective pressures (i.e., 0.3 or 1 µg/mL of histidine) are able to grow in the absence of the amino acid. 

To check this and to try to correlate the type of reversion occurred and the HisF protein structure with the ability to grow under different growth conditions, HisF^+^ revertants were streaked on MMD plates containing 0, 0.3, 1 and 25 µg/mL histidine. The data obtained (not shown) revealed that most of them were able to grow in the absence of histidine independently from the day of appearance on MMD plates and the selective pressure applied (see M.C.H. values reported in Table 1). On the contrary, revertants belonging to the last five groups (21–25, isolated in the presence of 0.3 or 1 µg/mL of histidine and exhibiting the +7, + 10 and −2 bp indels) were not able to grow in the total absence of the amino acid. 

**Figure 4:**
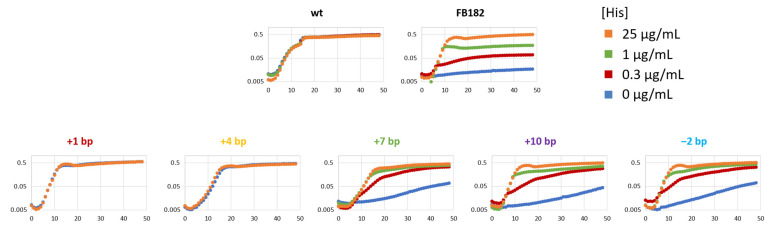
Growth curves of His+ revertants in absence and presence of histidine 0.3, 1 and 25 µg/mL. Hours of incubation are reported on the *x*-axis, while logarithm of O.D._600_ on the *y*-axis of the charts. Growth curves are reported for one representative of each indels group (+1 bp group: ID 14; +4 bp group: ID 19; +7 bp group: ID 21; +10 bp group: ID 22; −2 bp group: ID 24). Growth curves of all the single revertants are reported in Supplementary Material (Figure S1).

Thus, as described in Materials and Methods, growth curves in the absence and presence of histidine 25 µg/mL were performed for a representative of each of the 22 groups harboring a different HisF amino acid sequence (Figure 4 and Figure S1), confirming that +1 revertants were indistinguishable from the parental, and +4 revertants without histidine show slightly impaired growth curves, particularly types 16 and 17 whose amino acid sequence carries a higher number of residues different from the wild-type with respect to the other +4 mutants. The five revertants carrying a −2 bp deletion or +7 and +10 bp insertion were not able (or showed a very reduced ability) to grow in the complete absence of histidine, strongly suggesting that those mutations affected the correct functionality of the HisF protein. 

Hence, these five revertants were grown in MMD medium containing 0, 0.3, 1 and 25 µg/mL of histidine. The data obtained are reported in Figure 4 and Figure S1, whose analysis revealed that—as expected—the *E. coli* mutant FB182 did not grow in the absence of histidine but was able to grow slowly in the presence of 0.3 and 1 µg/mL of the amino acid, even if not reaching the final O.D._600_ typical of its growth in the presence of 25 µg/mL of histidine. The five mutants grew better than *E. coli* FB182 in the presence of 0.3 and 1 µg/mL of histidine, despite being slower than the wild-type strain. Without histidine, these revertants grew very slowly, but still better than the original mutant.

**Figure 5:**
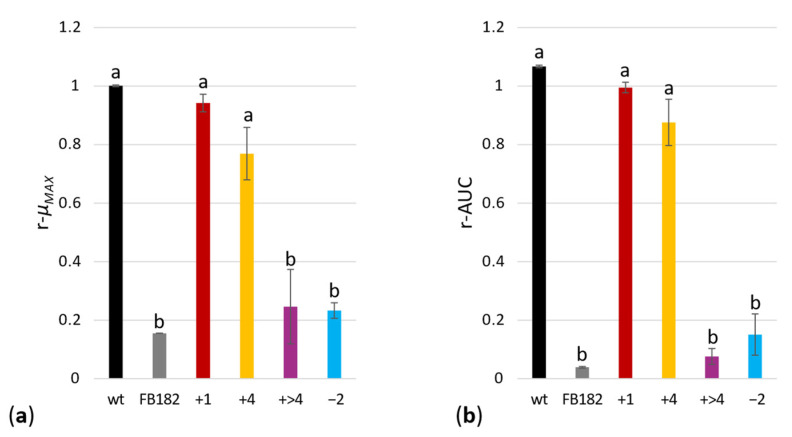
Average values of the ratios (**a**) between maximum growth rate (r-µ_MAX_) in absence and in presence of histidine, and (**b**) between area under growth curve (r-AUC) in absence and in presence of histidine. Each color corresponds to a distinct cell population (+7 bp and + 10 bp insertions were joined in the group called “+ >4”), whereas bars represent standard errors. Significant differences were evaluated through analysis of variance (ANOVA) performed using Tukey’s pairwise test. Different letters indicate significant differences (*p*-value < 0.001).

The results were confirmed by the analysis of the growth rate performed on the 22 HisF^+^ revertants and shown in Figure 5. 

Different scenarios can be depicted for this finding; the first one predicts that the catalytic activity of the HisF protein of revertants 21–25 was not completely recovered. As highlighted in Table 2 and Table 3, *E. coli* FB182 deletion lies in the phosphate-binding site, as well as all the revertant mutations. According to studies performed on *T. maritima* [44] and on the analysis of the HisF three-dimensional structures available in the Protein Data Bank (PDB) (PDB IDs: 1H5Y, 4EVZ, 5TQL, 7AC8), *E. coli* HisF^+^ revertant mutations did not change the residues directly involved in the phosphate binding, but fell immediately downstream of some of them (i.e., A231 and S232, Table 3). This makes sense, since mutations altering the amino acids directly binding the phosphate probably would not have allowed the functioning of the HisF protein and thus the growth of the revertants under selective pressure. However, some mutations could partially affect the correct functionality of the revertant HisF protein and, consequently, mutants’ ability to grow in the total absence of histidine in the culture medium. A second hypothesis relies on the fact that HisF can perform its activity when it interacts with the product of the *hisH* gene, forming the heterodimeric enzyme imidazole glycerol phosphate synthase (IGPS) [24]; hence, in principle, the slow growth of these revertants in the absence of histidine might be due to a weaker interaction of HisF protein with the HisH counterpart. However, at least in *T. maritima*, it is known that the HisH subunit docks onto the N-terminal face of the HisF subunit, located opposite the C-terminal’s active site face [45]. Since the revertant mutations are located in the C-terminal of HisF protein, its interaction with HisH subunit would appear not to be affected. A third possibility is based on the knowledge that IGPS catalyzes glutamine hydrolysis at the active site of HisH, producing ammonia (NH_3_) and glutamate, and that the generated NH_3_ travels from the HisH active site to the HisF one passing through the (β/α)_8_ barrel [46]. Thus, modifications in the three-dimensional HisF structure could affect the ammonia transfer from HisH to the C-terminal region of HisF. However, from the revertants HisF protein tertiary structure prediction, it was possible to observe that the modifications in the mutated enzymes conformation lie on the outer surface of the proteins, not affecting the HisF internal ammonia channel. A final hypothesis is based on the revertants HisF protein stability. Indeed, systematic studies of mutagenesis globally indicate that destabilization is the most common consequence of sequence alteration [47,48]. The revertants mutated amino acids are mainly located on the first turn (N-terminal) of the last α-helix. The C=O of the Asn side chain (N240 in the wild-type HisF protein) makes a typical H bond with the NH of the first turn of the helix. This is a typical helix N-cap and it is known to have a significant contribution to α-helix stability [49,50,51]. The alteration of this N-cap could determine the destabilization of some of the revertant HisF proteins. 

Thus, among these different hypotheses and based on the structural evidence, we could argue that the most likely ones are (i) the decreased binding affinity for phosphate, which, in turn, affects the catalytic activity without switching it off completely, and (ii) the destabilization of the HisF last α-helix.

### 3.8. Mechanisms of Frameshift Mutations

One of the typical mechanisms of frameshift mutation involves the insertion of one (or more) base(s), identical to an adjacent one already present in the original DNA [52]. This mechanism is represented by DNA polymerase slippage, occurring during DNA replication when DNA polymerase encounters a tandem repetition of single nucleotides or short oligonucleotides, and a transient dissociation of the template followed by an out-of-register annealing causes the pairing of a set of bases in one chain of the DNA molecule with the wrong, but complementary, set in the other chain. Studies have shown that the frequency of polymerase slippage increases as the number of repeat units in a run increases, with the most frequent types of corresponding frameshift mutations being single-unit insertions or deletions [53] and references therein.

It is possible that DNA polymerase slippage might be responsible for the generation of (see Table 2) (i) type 6 insertion where a single A has been added in the triplet AAA, (ii) type 15 insertion where slippage presumably caused both the substitution of a C with an A and the insertion of an additional adenine to the run of As, (iii) type 16 insertion, where the four nucleotides CAAA were inserted immediately downstream of two in-tandem CAAA repetitions, and lastly (iv) type 17 insertion, where the four nucleotides AAAA were inserted immediately downstream of a repetition of three As. A similar mechanism could have been involved in the generation of type 18 insertion, where the four nucleotides ATCA were inserted immediately downstream of another ATCA sequence. Moreover, aside from type 6 and 15 where the added nucleotides lie downstream of a run of at least three identical bases, it is possible to observe that in most of the other +1 bp insertion types (10 among 13), the inserted nucleotide is identical to one or two of the adjacent bases. In these cases, the number of units repetition is low, and the insertion of that specific nucleotide might be a coincidence; however, it is not possible to exclude that polymerase slippage could have been involved in at least some of these insertions. 

However, it is unlikely that all the obtained reversion mutations might have occurred through this mechanism, in particular those in which long sequences, different from the adjacent regions, were inserted (i.e., +7 and +10 bp insertions) or deleted. In addition to the polymerase slippage model, proposed for describing frameshift mutations in repetitive DNA, “misincorporation misalignment” and “dNTP-stabilized misalignment” models have been proposed for non-repetitive DNA. The first one occurs when DNA polymerase initially forms a mismatched base pair at the 3′-end that subsequently realigns by pairing with a complementary downstream template base prior to undergoing further extension. Alternatively, in the second case, DNA misalignment could occur as the first step followed by the “correct” incorporation of an incoming dNTP [54]. These models have been proposed mainly to explain frameshift deletions. 

## 4. Conclusions

In this work, we analyzed the molecular rearrangements responsible for the reversion of a deletion of a single nucleotide in the *E. coli hisF* mutant, trying to correlate the type of mutations occurred with: (i) the strength of the applied selective pressure, (ii) the time of appearance of reversions, (iii) the three-dimensional structure of the “reverted” HisF proteins, and (iv) the ability of these reverted proteins to perform the proper catalytic activity in the absence of histidine.

The data obtained revealed that a total of 67 indels of different lengths (+1, +4, +7, +10, and −2 bp) occurred, whose number decreased with the increase in the inserted nucleotides. Moreover, the lower the strength of the selective pressure, the higher the number of revertants appeared on selective medium, and an additional correlation was also observed between the type of the occurred rearrangement and the time requested for its appearance.

Some of the insertions were likely due to DNA polymerase slippage, whereas the molecular mechanisms responsible for most reversions (including the largest ones) are still unknown.

The finding that most of the indels occurred in a very narrow region, thus slightly modifying the amino acid sequence and the three-dimensional structure of the HisF protein, likely correlates to the necessity of a specific tertiary structure of the HisF protein to allow its correct functionality; the structural evidence of the different mutants is consistent with the respective ability to grow in the absence of histidine, confirming a correlation between the mutations and the catalytic activity of the enzyme. Given the lack of studies on this type of mutations and their effect on protein structure, it is difficult to say whether this phenomenon is generalized. In spite of this, the data reported in this work represent a further step toward the comprehension of the relation existing between changes in a gene sequence, the structure of the encoded protein and the phenotypic effects they have.

## Figures and Tables

**Table 1:** List of *E. coli* FB182 HisF^+^ revertants. The day of appearance on MMD containing different concentrations of histidine (0, 0.3 and 1 µg/mL) and the minimum concentration of histidine (M.C.H.) allowing the growth of revertants (see below) are reported. The ID represents the different type of molecular rearrangement occurred (see also Table 2).

ID	Mutant Name	Day of Appearance	[His] (µg/mL)	M.C.H.	Total	ID	Mutant Name	Day of Appearance	[His] (µg/mL)	M.C.H.	Total
**1**	24	8	1	0	2	**10**	G	6	0.3	0	3
	PC2	2	0.3	0			A1	2	0.3	0	
**2**	F16	12	1	0	4		3	2	0.3	0	
	A2	8	0.3	0		**11**	F03B	10	0	0	2
	F1A3	24	1	0			10	6	1	0	
	F03B2	14	0.3	0		**12**	I	2	1	0	6
**3**	F1B2	2	1	0	1		PHC1	2	0	0	
**4**	N	2	0	0	11		FP1B2	9	1	0	
	PHC15	11	1	0			2	4	0	0	
	F0B4	18	0	0			22	8	1	0	
	F0B5	22	0	0			A1_2	2	0.3	0	
	6	6	0.3	0		**13**	PHA1	3	0.3	0	1
	7	6	0.3	0		**14**	H	2	1	0	5
	8	2	1	0			C2	6	1	0	
	12	6	1	0			F1B3	7	1	0	
	18	8	1	0			F0B7	31	0	0	
	20	11	1	0			4	2	0.3	0	
	21	14	1	0		**15**	D1	3	1	0	1
**5**	F1	2	1	0	2	**16**	PB1	4	1	0	3
	FP03B1	2	0.3	0			9	3	1	0	
**6**	E	6	1	0	8		11	3	1	0	
	F	2	0.3	0		**17**	C1	6	1	0	1
	D1_2	3	1	0		**18**	C2_2	2	0.3	0	3
	F0B6	26	0	0			F03A1	2	0.3	0	
	13	7	1	0			17	11	1	0	
	15	11	0.3	0		**19**	PHA4	8	0.3	0	1
	16	15	0.3	0		**20**	C1_2	2	1	0	1
	19	8	1	0		**21**	PHB1	4	1	0.3	1
**7**	A2_2	2	0.3	0	1	**22**	F1A6	29	1	0.3	1
**8**	F0A1	2	0	0	3	**23**	F1A1	14	1	1	1
	F0A2	2	0	0		**24**	F1A4	29	1	1	1
	1	4	0	0		**25**	F1A2	20	1	0.3	2
**9**	F1B1	2	1	0	2		F03B1	13	0.3	0.3	
	F0AP1	2	1	0		**Total**					67

**Table 2:** Nucleotide sequence of *hisF* gene from *E. coli* FB182 HisF^+^ revertants. The type of mutation (I: insertion, D: deletion, S: substitution), the number of nucleotides inserted (+)/deleted (−), the location (u: upstream, d: downstream of the *E. coli* FB182 deletion) and position of the mutation, the number of revertants with a specific mutation (N. rev. A) and the number of revertants with the same number of inserted/deleted nucleotides (N. rev. B) are reported. The mutation is highlighted in red, the location of *E. coli* FB182 deletion is highlighted in grey. When mutation could have occurred in multiple positions, the last nucleotide(s) of the series is (are) highlighted and all the possible positions are reported. In the wild-type nucleotide sequence, the region belonging to the phosphate-binding domain is underlined. Different colors are assigned to the IDs on the basis of the number of inserted/deleted nucleotides. The same table reporting the reding frame for each sequence and the nucleotide sequence translation is reported in (Supplementary Material Table S1).

ID	Mut. Type	N. nt	Location	Position	Nucleotide Sequence (Positions 697–748)	N. Rev. A	N. Rev. B
**wt**	-	-	-	-	…GTATTCCACAAACAAATAATCAATATTGGTGAATTAAAAGCGTACCTGGCAA…	-	
**FB182**	D	−1	-	718–719	…GTATTCCACAAACAAATAATCA-TATTGGTGAATTAAAAGCGTACCTGGCAA…	-	
**1**	I	+1	-	718–719	…GTATTCCACAAACAAATAATCA**A**TATTGGTGAATTAAAAGCGTACCTGGCAA…	2	52
**2**	I	+1	u	717–718	…GTATTCCACAAACAAATAATC**C**A-TATTGGTGAATTAAAAGCGTACCTGGCAA…	4	
**3**	I	+1	u	716–717	…GTATTCCACAAACAAATAAT**T**CA-TATTGGTGAATTAAAAGCGTACCTGGCAA…	1	
**4**	I	+1	u	714–716	…GTATTCCACAAACAAATAA**A**TCA-TATTGGTGAATTAAAAGCGTACCTGGCAA…	11	
**5**	I	+1	u	713–714	…GTATTCCACAAACAAAT**T**AATCA-TATTGGTGAATTAAAAGCGTACCTGGCAA…	2	
**6**	I	+1	u	710–713	…GTATTCCACAAACAAA**A**TAATCA-TATTGGTGAATTAAAAGCGTACCTGGCAA…	8	
**7**	I	+1	d	720	…GTATTCCACAAACAAATAATCA-T**G**ATTGGTGAATTAAAAGCGTACCTGGCAA…	1	
**8**	I	+1	d	721	…GTATTCCACAAACAAATAATCA-TA**C**TTGGTGAATTAAAAGCGTACCTGGCAA…	3	
**9**	I	+1	d	721–723	…GTATTCCACAAACAAATAATCA-TATT**T**GGTGAATTAAAAGCGTACCTGGCAA…	2	
**10**	I	+1	d	720–721	…GTATTCCACAAACAAATAATCA-TA**A**TTGGTGAATTAAAAGCGTACCTGGCAA…	3	
**11**	I	+1	d	723–725	…GTATTCCACAAACAAATAATCA-TATTGG**G**TGAATTAAAAGCGTACCTGGCAA…	2	
**12**	I	+1	d	727–729	…GTATTCCACAAACAAATAATCA-TATTGGTGAA**A**TTAAAAGCGTACCTGGCAA…	6	
**13**	I	+1	d	728	…GTATTCCACAAACAAATAATCA-TATTGGTGA**T**ATTAAAAGCGTACCTGGCAA…	1	
**14**	I	+1	d	729–731	…GTATTCCACAAACAAATAATCA-TATTGGTGAATT**T**AAAAGCGTACCTGGCAA…	5	
**15**	S + I	+1	u	709 + 706–713	…GTATTCCACAAA**A**AAA**A**TAATCA-TATTGGTGAATTAAAAGCGTACCTGGCAA…	1	
**16**	I	+4	u	705/709/713	…GTATTCCACAAACAAA**CAAA**TAATCA-TATTGGTGAATTAAAAGCGTACCTGGCAA…	3	9
**17**	I	+4	u	711–713	…GTATTCCACAAACAAA**AAAA**TAATCA-TATTGGTGAATTAAAAGCGTACCTGGCAA…	1	
**18**	I	+4	d	715/719	…GTATTCCACAAACAAATAATCA-**ATCA**TATTGGTGAATTAAAAGCGTACCTGGCAA…	3	
**19**	I	+4	d	719	…GTATTCCACAAACAAATAATCA-**TACA**TATTGGTGAATTAAAAGCGTACCTGGCAA…	1	
**20**	I	+4	d	721	…GTATTCCACAAACAAATAATCA-TA**TTTA**TTGGTGAATTAAAAGCGTACCTGGCAA…	1	
**21**	I	+7	u	702	…GTATT**CCACATT**CCACAAACAAATAATCA-TATTGGTGAATTAAAAGCGTACCTGGCAA…	1	1
**22**	I	+10	d	719	…GTATTCCACAAACAAATAATCA-**TATTGAATCA**TATTGGTGAATTAAAAGCGTACCTGGCAA…	1	1
**23**	D	−2	u	713–714	…GTATTCCACAAACAAA**--**ATCA-TATTGGTGAATTAAAAGCGTACCTGGCAA…	1	4
**24**	D	−2	u	717–718	…GTATTCCACAAACAAATAAT**--**-TATTGGTGAATTAAAAGCGTACCTGGCAA…	1	
**25**	D	−2	d	723–724	…GTATTCCACAAACAAATAATCA-TATT**--**TGAATTAAAAGCGTACCTGGCAA…	2	

**Table 3:** Predicted amino acid sequence of the C-terminal region of HisF protein from *E. coli* FB182 HisF^+^ revertants. The type of mutation (I: insertion, D: deletion, S: substitution), the number of nucleotides inserted (+)/deleted (−), the location (u: upstream, d: downstream of the *E. coli* FB182 deletion) and position of the mutation, the number of revertants with a specific nucleotide mutation (N. rev. A), the number of revertants with the same amino acid sequence (N. rev. B) and the number of revertants with the same number of inserted/deleted nucleotides (N. rev. C) are reported. The asterisks represent the end of the sequence. The different amino acids are highlighted in red. In the wild-type amino acid sequence, the region belonging to the phosphate-binding domain [36] is underlined and two of the residues directly involved in the phosphate binding [42] are in bold. Different colors are assigned to the IDs on the basis of the number of inserted/deleted nucleotides.

ID	Mut. Type	N. nt	Location	Position	Amino Acid Sequence (Positions 229–258)	N. Rev. A	N. Rev. B	N. Rev. C
**wt**	-	-	-	-	…LA**AS**VFHKQIINIGELKAYLATQGVEIRIC*	-	-	-
**FB182**	D	−1	-	718–719	…LAASVFHKQIIILVN*	-	-	-
**1**	I	+1	-	718–719	…LAASVFHKQII**N**IGELKAYLATQGVEIRIC*	2	2	52
**2**	I	+1	u	717–718	…LAASVFHKQII**H**IGELKAYLATQGVEIRIC*	4	5	
**3**	I	+1	u	716–717	…LAASVFHKQII**H**IGELKAYLATQGVEIRIC*	1		
**4**	I	+1	u	714–716	…LAASVFHKQI**NH**IGELKAYLATQGVEIRIC*	11	13	
**5**	I	+1	u	713–714	…LAASVFHKQI**NH**IGELKAYLATQGVEIRIC*	2		
**6**	I	+1	u	710–713	…LAASVFHKQ**NNH**IGELKAYLATQGVEIRIC*	8	8	
**7**	I	+1	d	720	…LAASVFHKQII**M**IGELKAYLATQGVEIRIC*	1	1	
**8**	I	+1	d	721	…LAASVFHKQII**IL**GELKAYLATQGVEIRIC*	3	5	
**11**	I	+1	d	721–723	…LAASVFHKQII**IL**GELKAYLATQGVEIRIC*	2		
**9**	I	+1	d	720–721	…LAASVFHKQII**IF**GELKAYLATQGVEIRIC*	2	2	
**10**	I	+1	d	723–725	…LAASVFHKQII**II**GELKAYLATQGVEIRIC*	3	3	
**12**	I	+1	d	727–729	…LAASVFHKQII**ILVK**LKAYLATQGVEIRIC*	6	6	
**13**	I	+1	d	728	…LAASVFHKQII**ILVI**LKAYLATQGVEIRIC*	1	1	
**14**	I	+1	d	729–731	…LAASVFHKQII**ILVN**LKAYLATQGVEIRIC*	5	5	
**15**	S + I	+1	u	709 + 706–713	…LAASVFHK**KNNH**IGELKAYLATQGVEIRIC*	1	1	
**16**	I	+4	u	705/709/713	…LAASVFHKQ**TNNH**IGELKAYLATQGVEIRIC*	3	3	9
**17**	I	+4	u	711–713	…LAASVFHKQ**KNNH**IGELKAYLATQGVEIRIC*	1	1	
**18**	I	+4	d	715/719	…LAASVFHKQIIN**H**IGELKAYLATQGVEIRIC*	3	3	
**19**	I	+4	d	719	…LAASVFHKQII**IH**IGELKAYLATQGVEIRIC*	1	1	
**20**	I	+4	d	721	…LAASVFHKQII**IF**IGELKAYLATQGVEIRIC*	1	1	
**21**	I	+7	u	702	…LAASVFH**IPQTNNH**IGELKAYLATQGVEIRIC*	1	1	1
**22**	I	+10	d	719	…LAASVFHKQII**ILNH**IGELKAYLATQGVEIRIC*	1	1	1
**23**	D	−2	u	713–714	…LAASVFHKQ**NH**IGELKAYLATQGVEIRIC*	1	1	4
**24**	D	−2	u	717–718	…LAASVFHKQII**I**GELKAYLATQGVEIRIC*	1	1	
**25**	D	−2	d	723–724	…LAASVFHKQII**IF**ELKAYLATQGVEIRIC*	2	2	

## Supplementary Materials

The following supporting information can be downloaded at: https://www.mdpi.com/article/10.3390/microorganisms10040692/s1, Table S1: Nucleotide and amino acid sequences of *hisF* gene and HisF proteins from *E. coli* FB182 HisF^+^ revertants; Figure S1: Growth curves of His^+^ revertants.

## References

[1] Dijkhuizen L., Roberts D.M., Sharp P., Alderson G., Collins M.A. (1996). Evolution of metabolic pathways. Evolution of Microbial Life.

[2] Hall B.G. (1999). Toward an understanding of evolutionary potential. FEMS Microbiol. Lett..

[3] Cairns J., Overbaugh J., Miller S. (1988). The origin of mutants. Nature.

[4] Hall B.G. (1990). Spontaneous point mutations that occur more often when advantageous than when neutral. Genetics.

[5] Foster P.L. (1999). Mechanisms of Stationary Phase Mutation: A Decade of Adaptive Mutation. Annu. Rev. Genet..

[6] Maisnier-Patin S., Roth J.R. (2015). The Origin of Mutants Under Selection: How Natural Selection Mimics Mutagenesis (Adaptive Mutation). Cold Spring Harb. Perspect. Biol..

[7] Stahl F.W. (1988). A unicorn in the garden. Nature.

[8] Davis B.D. (1989). Transcriptional bias: A non-Lamarckian mechanism for substrate-induced mutations. Proc. Natl. Acad. Sci. USA.

[9] Boe L. (1990). Mechanism for induction of adaptive mutations in Escherichia coli. Mol. Microbiol..

[10] Galitski T., Roth J.R. (1995). Evidence that F Plasmid Transfer Replication Underlies Apparent Adaptive Mutation. Science.

[11] Cairns J., Foster P. (1991). Adaptive reversion of a frameshift mutation in Escherichia coli. Genetics.

[12] Alonso A., Campanario E., Martínez J.L. (1999). Emergence of multidrug-resistant mutants is increased under antibiotic selective pressure in Pseudomonas aeruginosa. Microbiology.

[13] Perfeito L., Fernandes L., Mota C., Gordo I. (2007). Adaptive Mutations in Bacteria: High Rate and Small Effects. Science.

[14] Wong J.T.-F. (1983). Membership mutation of the genetic code: Loss of fitness by tryptophan. Proc. Natl. Acad. Sci. USA.

[15] Bacher J.M., Bull J.J., Ellington A.D. (2003). Evolution of phage with chemically ambiguous proteomes. BMC Evol. Biol..

[16] Hoesl M.G., Oehm M.S.S., Durkin P., Darmon E., Peil L., Aerni H., Rappsilber J., Rinehart J., Leach D., Söll D. (2015). Chemical Evolution of a Bacterial Proteome. Angew. Chem. Int. Ed..

[17] Agostini F., Sinn L., Petras D., Schipp C.J., Kubyshkin V., Berger A.A., Dorrestein P.C., Rappsilber J., Budisa N., Koksch B. (2020). Multiomics Analysis Provides Insight into the Laboratory Evolution of Escherichia coli toward the Metabolic Usage of Fluorinated Indoles. ACS Central Sci..

[18] Bridges B.A. (1994). Starvation-associated mutation in Escherichia coli: A spontaneous lesion hypothesis for “directed” mutation. Mutat. Res. Mol. Mech. Mutagen..

[19] Sikora A., Grzesiuk E. (2010). Reversion of argE3 to Arg(+) in Escherichia coli AB1157—An informative bacterial system for mutation detection. Acta Biochim. Pol..

[20] Ames B.N., McCann J., Yamasaki E. (1975). Methods for detecting carcinogens and mutagens with the salmonella/mammalian-microsome mutagenicity test. Mutat. Res. Environ. Mutagen. Relat. Subj..

[21] Prival M.J., A Cebula T. (1992). Sequence analysis of mutations arising during prolonged starvation of Salmonella typhimurium. Genetics.

[22] Gizatullin F.S., Babynin E. (1996). The selection-induced His+ reversion in Salmonella typhimurium. Mutat. Res. Mol. Mech. Mutagen..

[23] Goldschmidt E.P., Cater M.S., Matney T.S., Butler M.A., Greene A. (1970). Genetic Analysis of the Histidine Operon in Escherichia Coli K12. Genetics.

[24] Chioccioli S., Bogani P., Del Duca S., Castronovo L.M., Vassallo A., Puglia A.M., Fani R. (2020). In vivo evaluation of the interaction between the Escherichia coli IGP synthase subunits using the Bacterial Two-Hybrid system. FEMS Microbiol. Lett..

[25] Gerlt J.A. (2000). New wine from old barrels. Nat. Genet..

[26] Copley R.R., Bork P. (2000). Homology among (βα)8 barrels: Implications for the evolution of metabolic pathways. J. Mol. Biol..

[27] Kasai T. (1974). Regulation of the expression of the histidine operon in Salmonella typhimurium. Nature.

[28] Davis B.D., Mingioli E.S. (1950). Mutants of Escherichia Coli Requiring Methionine or Vitamin B 12. J. Bacteriol..

[29] Sambrook J., Fritsch E.F., Maniatis T. (1989). Molecular Cloning: A Laboratory Manual.

[30] Hall T.A. (1999). BIOEDIT: A user-friendly biological sequence alignment editor and analysis program for Windows 95/98/ NT. Nucleic Acids Symp. Ser..

[31] Baek M., DiMaio F., Anishchenko I., Dauparas J., Ovchinnikov S., Lee G.R., Wang J., Cong Q., Kinch L.N., Schaeffer R.D. (2021). Accurate prediction of protein structures and interactions using a three-track neural network. Science.

[32] Jumper J., Evans R., Pritzel A., Green T., Figurnov M., Ronneberger O., Tunyasuvunakool K., Bates R., Žídek A., Potapenko A. (2021). Highly accurate protein structure prediction with AlphaFold. Nature.

[33] Pettersen E.F., Goddard T.D., Huang C.C., Couch G.S., Greenblatt D.M., Meng E.C., Ferrin T.E. (2004). UCSF Chimera—A visualization system for exploratory research and analysis. J. Comput. Chem..

[34] Widdel F. (2007). Theory and Measurement of Bacterial Growth A. Basic and practical aspects. Dalam Grund. Mikrobiol..

[35] Atkinson K.E. (1990). An Introduction to Numerical Analysis. Math. Comput..

[36] Bork P., Gellerich J., Groth H., Hooft R., Martin F. (1995). Divergent evolution of a β/α-barrel subclass: Detection of numerous phosphate-binding sites by motif search. Protein Sci..

[37] Lang D., Thoma R., Henn-Sax M., Sterner R., Wilmanns M. (2000). Structural Evidence for Evolution of the β/α Barrel Scaffold by Gene Duplication and Fusion. Science.

[38] Wierenga R. (2001). The TIM-barrel fold: A versatile framework for efficient enzymes. FEBS Lett..

[39] Reisinger B., Sperl J., Holinski A., Schmid V., Rajendran C., Carstensen L., Schlee S., Blanquart S., Merkl R., Sterner R. (2013). Evidence for the Existence of Elaborate Enzyme Complexes in the Paleoarchean Era. J. Am. Chem. Soc..

[40] Nagano N., A Orengo C., Thornton J. (2002). One Fold with Many Functions: The Evolutionary Relationships between TIM Barrel Families Based on their Sequences, Structures and Functions. J. Mol. Biol..

[41] Foster P.L. (2007). Stress-Induced Mutagenesis in Bacteria. Crit. Rev. Biochem. Mol. Biol..

[42] Beismann-Driemeyer S., Sterner R. (2001). Imidazole Glycerol Phosphate Synthase from Thermotoga maritima. J. Biol. Chem..

[43] Eisenstein M. (2021). Artificial intelligence powers protein-folding predictions. Nature.

[44] Liu S., Wu C., Chen C. (2022). The computational models of AlphaFold2 and RoseTTAfold carry protein foldability information. bioRxiv.

[45] Douangamath A., Walker M., Beismann-Driemeyer S., Vega-Fernandez M., Sterner R., Wilmanns M. (2002). Structural Evidence for Ammonia Tunneling across the (βα)8 Barrel of the Imidazole Glycerol Phosphate Synthase Bienzyme Complex. Structure.

[46] Rivalta I., Lisi G.P., Snoeberger N.-S., Manley G., Loria J.P., Batista V.S. (2016). Allosteric Communication Disrupted by a Small Molecule Binding to the Imidazole Glycerol Phosphate Synthase Protein–Protein Interface. Biochemistry.

[47] Colacino J.M., Chirgadze N.Y., Garman E., Murti K., Loncharich R.J., Baxter A.J., A Staschke K., Laver W. (1997). A Single Sequence Change Destabilizes the Influenza Virus Neuraminidase Tetramer. Virology.

[48] Gyulkhandanyan A., Rezaie A.R., Roumenina L., Lagarde N., Fremeaux-Bacchi V., Miteva M., Villoutreix B.O. (2020). Analysis of protein missense alterations by combining sequence- and structure-based methods. Mol. Genet. Genom. Med..

[49] Muñoz V., Serrano L. (1995). Helix design, prediction and stability. Curr. Opin. Biotechnol..

[50] Harpaz Y., Elmasry N., Fersht A.R., Henrick K. (1994). Direct observation of better hydration at the N terminus of an alpha-helix with glycine rather than alanine as the N-cap residue. Proc. Natl. Acad. Sci. USA.

[51] Thapar R., Nicholson E.M., Rajagopal P., Waygood E.B., Scholtz J.M., Klevit R.E. (1996). Influence of N-Cap Mutations on the Structure and Stability of Escherichia coli HPr. Biochemistry.

[52] Streisinger G., Okada Y., Emrich J., Newton J., Tsugita A., Terzaghi E., Inouye M. (1966). Frameshift Mutations and the Genetic Code. Cold Spring Harb. Symp. Quant. Biol..

[53] Gragg H., Harfe B.D., Jinks-Robertson S. (2002). Base Composition of Mononucleotide Runs Affects DNA Polymerase Slippage and Removal of Frameshift Intermediates by Mismatch Repair in *Saccharomyces cerevisiae*. Mol. Cell. Biol..

[54] Tippin B., Kobayashi S., Bertram J.G., Goodman M.F. (2004). To Slip or Skip, Visualizing Frameshift Mutation Dynamics for Error-prone DNA Polymerases. J. Biol. Chem..

