# Supplementary material for: Effect of Non-Lethal Selection on Spontaneous Revertants of Frameshift Mutations: The Escherichia coli hisF Case
Source: Microorganisms. 2022 Mar 23;10(4):692. doi: 10.3390/microorganisms10040692 (PMC9032791; doi:10.3390/microorganisms10040692)
Supplement: Supplementary file 1 [file microorganisms-10-00692-s001.zip › microorganisms-1619600-supplementary.pdf]

Table S1. Nucleotide and amino acid sequences of *hisF* gene and HisF proteins, respectively, from *E. coli* FB182 HisF+ revertants. The reading frame of the nucleotide sequences is highlighted. The type of mutation (I: insertion, D: deletion, S: substitution), the number of nucleotides inserted (+)/deleted (-), the location (u: upstream, d: downstream of the *E. coli* FB182 deletion) and position of the mutation, the number of revertants with a specific mutation (N. rev. A) and the number of revertants with the same number of inserted/deleted nucleotides (N. rev. B) are reported. The mutation is highlighted in red, the location of *E. coli* FB182 deletion is highlighted in grey. When mutation could have occurred in multiple positions, the last nucleotide(s) of the series is (are) highlighted and all the possible positions are reported. In the wt nucleotide sequence, the region belonging to the phosphate-binding domain is underlined. Different colors are assigned to the IDs on the basis of the number of inserted/deleted nucleotides.

ID	Mut. type	N. nt	Location	Position	Nucleotide sequence (positions 697-748)	N. rev.A	N. rev.B
**wt**	-	-	-	-	...GTA.TTC.CAC.AAA.CAA.ATA.ATC.AAT.ATT.GGT.GAA.TTA.AAA.GCG.TAC.CTG.GCA.A...<br>V F H K Q I I N I G E L K A Y L A	-	
**FB182**	D	-1	-	718-719	...GTA.TTC.CAC.AAA.CAA.ATA.ATC.A-TA.TTG.GTG.AAT.TAA.AAG.CGT.ACC.TGG.CAA...<br>V F H K Q I I I L V N *	-	
1	I	+1	-	718-719	...GTA.TTC.CAC.AAA.CAA.ATA.ATC.AAT.ATT.GGT.GAA.TTA.AAA.GCG.TAC.CTG.GCA.A...<br>V F H K Q I I N I G E L K A Y L A	2	
2	I	+1	u	717-718	...GTA.TTC.CAC.AAA.CAA.ATA.ATC.CA-T.ATT.GGT.GAA.TTA.AAA.GCG.TAC.CTG.GCA.A...<br>V F H K Q I I H I G E L K A Y L A	4	
3	I	+1	u	716-717	...GTA.TTC.CAC.AAA.CAA.ATA.ATT.CA-T.ATT.GGT.GAA.TTA.AAA.GCG.TAC.CTG.GCA.A...<br>V F H K Q I I H I G E L K A Y L A	1	
4	I	+1	u	714-716	...GTA.TTC.CAC.AAA.CAA.ATA.AAT.CA-T.ATT.GGT.GAA.TTA.AAA.GCG.TAC.CTG.GCA.A...<br>V F H K Q I N H I G E L K A Y L A	11	
5	I	+1	u	713-714	...GTA.TTC.CAC.AAA.CAA.ATT.AAT.CA-T.ATT.GGT.GAA.TTA.AAA.GCG.TAC.CTG.GCA.A...<br>V F H K Q I N H I G E L K A Y L A	2	
6	I	+1	u	710-713	...GTA.TTC.CAC.AAA.CAA.AAT.AAT.CA-T.ATT.GGT.GAA.TTA.AAA.GCG.TAC.CTG.GCA.A...<br>V F H K Q N N H I G E L K A Y L A	8	
7	I	+1	d	720	...GTA.TTC.CAC.AAA.CAA.ATA.ATC.A-TG.ATT.GGT.GAA.TTA.AAA.GCG.TAC.CTG.GCA.A...<br>V F H K Q I I M I G E L K A Y L A	1	
8	I	+1	d	721	...GTA.TTC.CAC.AAA.CAA.ATA.ATC.A-TA.CTT.GGT.GAA.TTA.AAA.GCG.TAC.CTG.GCA.A...<br>V F H K Q I I I L G E L K A Y L A	3	52
9	I	+1	d	721-723	...GTA.TTC.CAC.AAA.CAA.ATA.ATC.A-TA.TTT.GGT.GAA.TTA.AAA.GCG.TAC.CTG.GCA.A...<br>V F H K Q I I I F G E L K A Y L A	2	
10	I	+1	d	720-721	...GTA.TTC.CAC.AAA.CAA.ATA.ATC.A-TA.ATT.GGT.GAA.TTA.AAA.GCG.TAC.CTG.GCA.A...<br>V F H K Q I I I I G E L K A Y L A	3	
11	I	+1	d	723-725	...GTA.TTC.CAC.AAA.CAA.ATA.ATC.A-TA.TTG.GGT.GAA.TTA.AAA.GCG.TAC.CTG.GCA.A...<br>V F H K Q I I I L G E L K A Y L A	2	
12	I	+1	d	727-729	...GTA.TTC.CAC.AAA.CAA.ATA.ATC.A-TA.TTG.GTG.AAA.TTA.AAA.GCG.TAC.CTG.GCA.A...<br>V F H K Q I I I L V K L K A Y L A	6	
13	I	+1	d	728	...GTA.TTC.CAC.AAA.CAA.ATA.ATC.A-TA.TTG.GTG.ATA.TTA.AAA.GCG.TAC.CTG.GCA.A...<br>V F H K Q I I I L V I L K A Y L A	1	
14	I	+1	d	729-731	...GTA.TTC.CAC.AAA.CAA.ATA.ATC.A-TA.TTG.GTG.AAT.TTA.AAA.GCG.TAC.CTG.GCA.A...<br>V F H K Q I I I L V N L K A Y L A	5	
15	S + I	+1	u	709 + 706-713	...GTA.TTC.CAC.AAA.AAA.AAT.AAT.CA-T.ATT.GGT.GAA.TTA.AAA.GCG.TAC.CTG.GCA.A...<br>V F H K K N N H I G E L K A Y L A	1	

16	I	+4	u	705/709/713	…GTA.TTC.CAC.AAA.CAA.ACA.AAT.AAT.CA-T.ATT.GGT.GAA.TTA.AAA.GCG.TAC.CTG.GCA.A…<br>V F H K Q T N N H I G E L K A Y L A	3	
17	I	+4	u	711-713	…GTA.TTC.CAC.AAA.CAA.AAA.AAT.AAT.CA-T.ATT.GGT.GAA.TTA.AAA.GCG.TAC.CTG.GCA.A…<br>V F H K Q K N N H I G E L K A Y L A	1	
18	I	+4	d	715/719	…GTA.TTC.CAC.AAA.CAA.ATA.ATC.A-AT.CAT.ATT.GGT.GAA.TTA.AAA.GCG.TAC.CTG.GCA.A…<br>V F H K Q I I N H I G E L K A Y L A	3	9
19	I	+4	d	719	…GTA.TTC.CAC.AAA.CAA.ATA.ATC.A-TA.CAT.ATT.GGT.GAA.TTA.AAA.GCG.TAC.CTG.GCA.A…<br>V F H K Q I I I H I G E L K A Y L A	1	
20	I	+4	d	721	…GTA.TTC.CAC.AAA.CAA.ATA.ATC.A-TA.TTT.ATT.GGT.GAA.TTA.AAA.GCG.TAC.CTG.GCA.A…<br>V F H K Q I I I F I G E L K A Y L A	1	
21	I	+7	u	702	…GTA.TTC.CAC.ATT.CCA.CAA.ACA.AAT.AAT.CA-T.ATT.GGT.GAA.TTA.AAA.GCG.TAC.CTG.GCA.A…<br>V F H I P Q T N N H I G E L K A Y L A	1	1
22	I	+10	d	719	…GTA.TTC.CAC.AAA.CAA.ATA.ATC.A-TA.TTG.AAT.CAT.ATT.GGT.GAA.TTA.AAA.GCG.TAC.CTG.GCA.A…<br>V F H K Q I I I L N H I G E L K A Y L A	1	1
23	D	-2	u	713-714	…GTA.TTC.CAC.AAA.CAA.A--AT.CA-T.ATT.GGT.GAA.TTA.AAA.GCG.TAC.CTG.GCA.A…<br>V F H K Q N H I G E L K A Y L A	1	
24	D	-2	u	717-718	…GTA.TTC.CAC.AAA.CAA.ATA.AT---T.ATT.GGT.GAA.TTA.AAA.GCG.TAC.CTG.GCA.A…<br>V F H K Q I I I G E L K A Y L A	1	4
25	D	-2	d	723-724	…GTA.TTC.CAC.AAA.CAA.ATA.ATC.A-TA.TT--T.GAA.TTA.AAA.GCG.TAC.CTG.GCA.A…<br>V F H K Q I I I F E L K A Y L A	2	

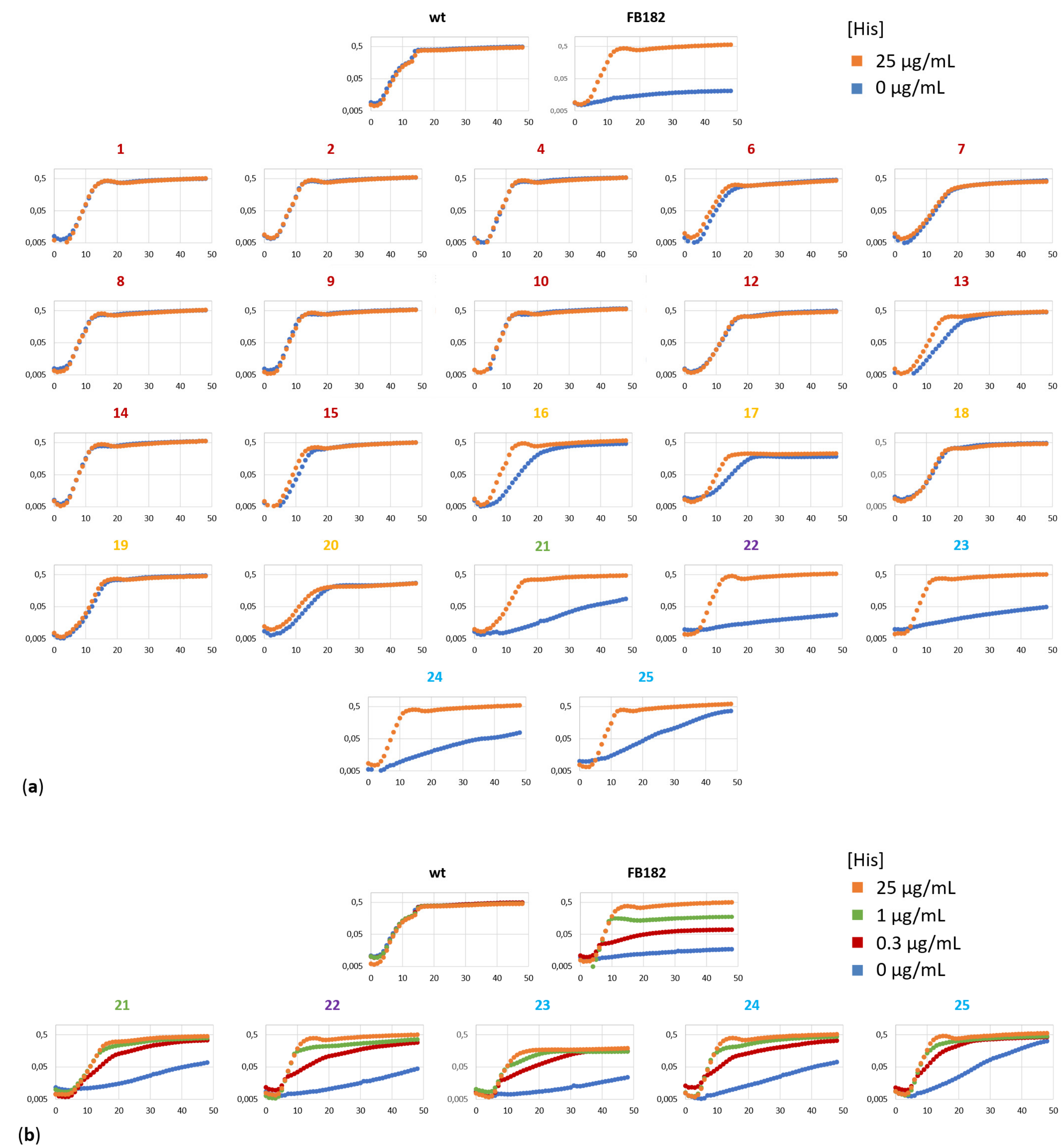

Figure S1. Growth curves of His+ revertants **(a)** in absence and in presence of histidine 25 µg/mL, and **(b)** in absence and in presence of histidine 0.3, 1 and 25 µg/mL. Hours of incubation are reported on the x-axis, while logarithm of O.D.$_{600}$ on the y-axis of the charts.
